# Assessing Cranial Nerves in Physical Therapy Practice: Findings from a Cross-Sectional Survey and Implication for Clinical Practice

**DOI:** 10.3390/healthcare9101262

**Published:** 2021-09-24

**Authors:** Firas Mourad, Giovanni Lopez, Fabio Cataldi, Filippo Maselli, Leonardo Pellicciari, Mattia Salomon, Hendrikus Kranenburg, Roger Kerry, Alan Taylor, Nathan Hutting

**Affiliations:** 1 Department of Physiotherapy, LUNEX International University of Health, Exercise and Sports, 4671 Differdange, Luxembourg; firas.mourad@me.com; 2 Luxembourg Health & Sport Sciences Research Institute A.s.b.l., 50, Avenue du Parc des Sports, 4671 Differdange, Luxembourg; 3 Department of Clinical Science and Translation Medicine, Faculty of Medicine and Surgery, University of Rome Tor Vergata, 00133 Roma, Italy; giolop@gmail.com (G.L.); fabio.cataldi@gmail.com (F.C.); salomon.mattia@gmail.com (M.S.); 4 Department of Physiotherapy, Kinesis, 70126 Bari, Italy; 5 Department of Physiotherapy, Manual Therapy Laboratory—MTLab, 70123 Bari, Italy; 6 Sovrintendenza Sanitaria Regionale Puglia INAIL, 70126 Bari, Italy; 7 Department of Neurosciences, Rehabilitation, Ophthalmology, Genetic and Maternal Infantile Sciences (DINOGMI), Campus of Savona, University of Genova, 16132 Savona, Italy; 8 IRCCS Fondazione Don Carlo Gnocchi, 50143 Florence, Italy; leonardo.pellicciari@gmail.com; 9 Department of Physical Therapy, Centro Diagnostico e Polispecialistico CST S.r.l., 38121 Trento, Italy; 10 Research Group Healthy Ageing, Allied Health Care and Nursing, Hanze University of Applied Sciences, 9714 CE Groningen, The Netherlands; h.a.kranenburg@pl.hanze.nl; 11 Division of Physiotherapy and Sport Rehabilitation, School of Health Sciences, Faculty of Medicine and Health Sciences, Nottingham University, Nottingham NG5 1PB, UK; roger.kerry@nottingham.ac.uk (R.K.); alan.taylor@nottingham.ac.uk (A.T.); 12 Department of Occupation and Health, School of Organisation and Development, HAN University of Applied Sciences, 6503 GL Nijmegen, The Netherlands; Nathan.Hutting@han.nl

**Keywords:** cranial nerve examination, physical therapy, differential diagnosis, neck pain, neurological examination

## Abstract

Background and objective: Serious pathologies of the neck can potentially result in cranial nerve palsy. Knowledge about cranial nerve examination (CNE) seems sparse, and its use is still unknown. We aim to investigate the knowledge, skills, and utilization of CNE of Italian physiotherapists. Materials and Methods: An online cross-sectional survey. Results: 396 completed the survey, reaching the required sample size. Although Italian physiotherapists consider CNE relevant (mean ± SD = 7.6/10 ± 2.0), over half of all responders (*n* = 229 (57.8%)) were not trained in the fundamentals and around a third did not use it in their daily practice (*n* = 138 (34.8%)). Additionally, participants were unconfident and insecure in conducting (*n* = 152 (38.4%) and *n* = 147 (37.1%)), interpreting (*n* = 140 (35.4%) and *n* = 164 (41.4%)), and managing the CNE (*n* = 141 (35.6%) and *n* = 154 (38.9%)). Possessing a musculoskeletal specialization was associated with an increased value attributed to clinical practice guidelines and reduced the lack of confidence in conducting, interpreting, and managing the CNE (respectively, *n* = 35 (25.5%), *p* = 0.0001; *n* = 32 (23.4%) *p* = 0.0002; *n* = 32 (23.4%) *p* = 0.0002). Working in a direct access setting significantly increased the considered relevance of guidelines and the concerns about arterial (*p* = 0.004) and other serious pathologies (*p* = 0.021). Pain and visual disturbances were considered the main indicators to CNE, demonstrating limited knowledge of signs and symptoms’ indicating CNE. Participants considered specific training in CNE as relevant (mean ± SD = 7.6/10 = 2.1). Conclusions: a substantial proportion of Italian physiotherapists are not schooled in the fundamentals of cranial nerve examination. Given the number of physiotherapists who work in first contact roles, this is a professional concern.

## 1. Introduction

Neck pain and associated disorders (NAD) are common complex biopsychosocial disorders with a high physical, psychosocial, and economic impact, leading to increased healthcare utilization [1,2,3]. The Neck Pain Task Force recommends a four-grade classification system of neck pain severity that is intended to help patients, researchers, clinicians, and policy makers in framing their questions and decisions [4].

Clinical practice guidelines recommend ruling out signs or symptoms of major structural pathologies (i.e., NAD IV)—such as congenital craniovertebral anomalies, cervical vascular pathologies, anatomical instabilities, and autonomic disorders—masquerading as neck pain before providing any evidence-based intervention [1,5,6]. The screening for the referral process in case of serious pathologies in physiotherapy—especially in a direct access setting—is a professional challenge. The incidence of delayed diagnosis of serious pathologies ranges from 5% to 20% in the cervical region [7], leading to a lack of recognition that may result in life-threatening consequences [8]. Red flags are signs and symptoms that should alert physiotherapists to consider carefully if the patient is within their scope of practice [1,9,10] and whether they need appropriate medical referral [1,11,12,13]. Commonly, patients with NAD IV present subtle transient antecedent neurological signs and symptoms or risk factors (i.e., acute onset of unusual headache or neck pain, recent trauma to the head or neck, and/or ischemic signs and symptoms, in younger people under 50 years) [14,15]. Clinicians should identify these during the subjective patient history and further verify during the neurological testing [8,16,17,18,19], especially by the use of cranial nerve examination (CNE) because serious pathologies of the neck can potentially result in cranial nerve (CN) palsy (especially CNs V, VI, VIII, IX, X, and XII) [11,20,21,22]. Physiotherapists therefore require skills in a wide range of neurological examination procedures required to screen all potential NAD IV clinical presentations [23].

Only 5% of clinicians routinely screen for red flags during initial assessment [6,8]. However, screening for serious pathology is a priority [1,5,6] and requires expertise in that field and a systematic approach [24]. In addition, information about physiotherapists’ knowledge of CNE is sparse and its use by physiotherapists is still unknown. Our survey aims to investigate the knowledge, skills, and utilization of CNE in a sample of Italian physiotherapists. This study aims to contribute to the knowledge base and discussion regarding potential future directions for the screening for referral process of NAD IV.

## 2. Methods

An online cross-sectional questionnaire survey was developed using the online platform Survey Monkey (SVMK Inc., San Mateo, USA) for Italian physiotherapists. The study is reported in line with the Checklist for Reporting Results of Internet Surveys (CHERRIES) [25] and the Strengthening the Reporting of Observational Studies in Epidemiology (STROBE) guidelines [26].

### 2.1. Survey Development

With the permission of the authors, to develop our version, we translated, modified the contents of, and culturally adapted into the Italian setting an unpublished recent survey conducted in the U.K. [27]. The survey was revised and adapted by two authors (native English and Italian specialized musculoskeletal physiotherapists with experience in education and research; FMo and AF). Then, the survey was piloted by six experienced Italian physiotherapists and physicians (AP, EG, FC, FMa, MG, and FP) for additional feedback on wording, response logic, and the fulfillment duration. The use of the original U.K. survey and the feedback provided by the pilot stage, respectively, strengthened the content and the face validity.

The survey was structured in three sections: the first section investigated demographic information, practice settings, and the education level; the second investigated the knowledge, skills, and clinical impact of CNE; the last section investigated education and personal opinions.

The survey consisted of 36 questions with a combination of close-ended (few of them with multiple selection) and Likert-scale questions. Only one question was an open-ended question (Supplement 1). All questions were presented at the same order and were mandatory to complete the survey.

### 2.2. Setting and Recruitment

A web-link to the survey was distributed via a mailing list of the Italian Physiotherapists Association on 23 March 2020. To take advantage of the forced period due to the COVID-19 pandemic and to maximize the response rate, invitations to participate were frequently re-published once per week via social media networks (Facebook, Twitter, LinkedIn, and Instagram). The survey was open for one month, and the closing date was 26 April 2020. For pragmatic purposes and in line with previous internationally published surveys, we adopted this methodological approach with the aim to collect the maximum number of answers within a specific period as most responses occur early after posting [28,29,30,31,32,33,34,35]. A priori, a sample size was calculated using the e-survey Dillman’s formula [36] with a 95% confidence level and a 5% of margin of error. At the time of the survey, the number of physiotherapists registered to the Italian Physiotherapists Association was 7398; therefore, the required sample size for this study was 366 [37]. The questionnaire could be completed on any electronic device with internet access; as Survey Monkey was used without collecting respondents’ IP addresses, the recruitment was anonymous and voluntary; in addition, the same IP was not allowed to access to the survey more than one time. Completion took approximately 10–15 min. No compensation or reimbursement were offered.

### 2.3. Data Processing and Analysis

Data from the survey platform were transferred to, and stored in, an encrypted computer for the purposes of data analysis, and access was allowed only to researchers involved in the data analysis. Incomplete surveys were not collected nor analyzed. We analyzed the open-ended questions by coding and categorizing the answers, adopting a bottom-up mixed approach [38].

Descriptive statistics was computed to describe the collected variables. A Chi-squared independent test was run to study any difference between responses provided by sample subgroups (i.e., Orthopedic Manipulative Physical Therapist (OMPT) specialization, physiotherapy access regimen, and experience years) to the categorical questions; in case the Chi-squared revealed significant differences (*p* < 0.05), adjusted standardized residuals [39] with their Bonferroni-corrected *p*-value were calculated for each cell to identify which cells of the contingency tables were accountable for the significant effect [40,41]. Moreover, to study any differences between responses provided by sample subgroups to the ordinal (i.e., Likert) questions, an independent *t*-test or an univariate ANOVA with Bonferroni-corrected post-hoc comparisons was run for answers to two categories (i.e., OMPT specialization and physical therapy access regimen) or to five categories (i.e., experience years).

All statistical analyses were performed with SPSS software (SPSS. Version 20 for Windows; SPSS Inc., Chicago, IL, USA, 2004), and the level was set at *p*-value < 0.05 for all comparisons.

### 2.4. Ethics

This study was approved by the Human Subjects Committee of the Department of Physical Therapy, Occupational Therapy, Rehabilitation and Physical Medicine, Universidad Rey Juan Carlos of Madrid, with approval letter URJC—DPTO 55—2019. The authors followed the principles outlined in the Declaration of Helsinki for this study [42].

## 3. Results

### 3.1. Responses

A total of 420 physiotherapists provided the consent and completed the survey. Of those, 24 were excluded as they did not work in Italy and were not included in the final analysis with the purpose to avoid any bias affecting our findings [43]. A final number of 396 physiotherapists was included. Although available for a very short period, our sample was in line with previous Italian surveys and reached the required sample size [44,45].

### 3.2. Respondent Characteristics

A total of 137 (34.6%; 95%CI 29.9–39.3) physiotherapists possessed an OMPT (i.e., musculoskeletal specialization) by completing a university master’s program, following the International Federation of Orthopaedic Manipulative Physical Therapists’ standards. The majority of respondents worked in a primary line care (*n* = 314, 79.3%; 95%CI 75.3–83.3), and 41.4% (*n* = 164; 95%CI 36.6–46.3) worked in a direct setting regimen. Almost half had practiced for less than 10 years (*n* = 112, 28.3; 95%CI 23.8–32.7); of those, 24.5% practiced less than 5 years (*n* = 97; 95%CI 20.3–28.7). Further details are presented in Table 1.

### 3.3. Knowledge and Education

Although Italian physiotherapists attribute a moderate importance to guidelines for assessing NAD (mean = 7.5/10 points; SD = 2.1), most of them were not familiar with the utilization (*n* = 256, 64.6%; 95%CI 59.9–69.4). A significant difference was found in possessing an OMPT specialization and working in a direct setting in attributing importance to guidelines (*p* = 0.003). Notably, a moderate percentage declares to manage 1 to 5 patients per month with potentially concerning clinical presentations such as headache (*n* = 175, 44.2%; 95%CI 39.3–49.1), dizziness (*n* = 152, 38.4%; 95%CI 33.6–43.2), neck or head trauma (*n* = 143, 36.1%; 95%CI 31.4–40.8), and whiplash (*n* = 152, 38.4%; 95%CI 33.6–43.2) (Table 2).

Among the 396 respondents, only 167 (42.2%; 95%CI 37.3–47.0) reported having received information/training in the fundamentals of CNE mainly by personal reading (*n* = 83, 49.7%; 95%CI 44.8–54.6), continuing professional development courses (*n* = 77, 46.1%; 95%CI 38.5–53.7), and during a master’s program (*n* = 73, 43.7%; 95%CI 36.2–51.2). However, the majority of the respondents (*n* = 229, 57.8%; 95%CI 53.0–62.7) have not been sufficiently trained in CNE, primarily because of a lack of musculoskeletal focus during their undergraduate programs (*n* = 146, 63.8%; 95%CI 57.5–70.0). Interestingly, 71 (31.0%; 95%CI 25.0–37.0) did not feel the need to be educated in CNE because it was not considered relevant for their clinical practice (Table 3).

### 3.4. Knowledge, Confidence, and Skills in Conducting Cranial Nerve Examination

Overall, physiotherapists attributed high importance to CNE (mean = 7.6/10 points; SD = 2.0). A relevant number (*n* = 138, 34.8%; 30.2–39.5) reported that they did not use it in their clinical practice mainly because they were not trained adequately (*n* = 94, 68.1%; 95%CI 60.3–75.9). A total of 258 (65.2%; 95%CI 60.5–69.8) physiotherapists declared using CNE in their clinical practice; however, the majority included it in the patient’s physical examination rarely (*n* = 148, 57.4%; 95%CI 51.3–63.4) or occasionally (*n* = 86, 33.3%; 95%CI 27.6–39.1). A significant difference was found in those that rarely use the CNE and those with 0–5 years of practice (*n* = 44, 80%; *p* = 0.0001) (Table 4). The main items in the patient interview that prompt them to perform a CNE are visual disturbance (*n* = 101, 39.1%; 95%CI 33.1–45.1), pain (*n* = 101, 39.1%; 95%CI 33.1–45.1), sensibility changes (*n* = 88, 34.1%; 95%CI 28.3–39.8), and dizziness (*n* = 87, 33.7%; 95%CI 27.9–39.4). Importantly, the majority of all the participants felt unconfident (*n* = 152, 38.4%; 95%CI 33.6–43.2) or insecure (*n* = 147, 37.1%; 95%CI 32.4–41.9) in conducting a CNE; unconfident (*n* = 140, 35.4%; 95%CI 30.6–40.1) or insecure (*n* = 164, 41.4%; 95%CI 36.6–46.3) in interpreting the findings; and unconfident (*n* = 141, 35.6%; 95%CI 30.9–40.3) or insecure (*n* = 154, 38.9%; 95%CI 34.1–43.7) in managing the examination results.

A significant difference was found in the confidence in conducting CNE among participants (*p* < 0.001). Post hoc tests illustrated the differences between those with an OMPT qualification and the reduction of lack of confidence in the ability of conducting (*n* = 35, 25.5%; *p* = 0.0001), interpreting the findings (*n* = 32, 23.4%; *p* = 0.0002), and managing the abnormal findings (*n* = 32, 23.4%; *p* = 0.0002) (Table 2). Additionally, post hoc tests illustrated that those with 0–5 years of experience are significantly less sure in interpreting the findings (*n* = 11, 11.3%; *p* = 0.002) (Table 4). When abnormal findings were detected during the CNE, one-third (*n* = 128, 32.3%; 95%CI 27.7–36.9) of all respondents did not feel the need to refer to a physician in case of a positive finding during CNE, suggesting an underestimate of its clinical relevance.

### 3.5. Attitudes towards Instability and Cervical Arterial Pathologies

Respondents attributed moderate importance to CNE when assessing patients with potential cervical arterial pathologies (mean = 7.5/10 points; SD = 2.2) but less importance for cervical instability (i.e., congenital craniovertebral anomalies, cervical fractures, craniovertebral junction ligaments damage, etc.) (mean = 6.7/10 points; SD = 2.5). The most common used screening clinical tools for serious cervical conditions were the pre-manipulative tests (for both vertebro-basilar insufficiency and ligamentous instability) (*n* = 176, 44.4%; 95%CI 39.6–49.3). Notably, only 8.1% (*n* = 32; 95%CI = 5.4–10.8) and 11.6% (*n* = 46; 95%CI 8.5–14.8) use, respectively, the Canadian Cervical Spine Rules and meaningful items from history taking.

Generally, manual therapy—especially spinal thrust manipulation—was not considered directly linked or the direct cause of cervical arterial dissection (mean = 4.0/10 points; SD = 2.9). A significant difference was found in considering manual therapy to the neck causing adverse events in those with >20 years of experience (5.31 ± 3.15; *p* = 0.001) and in those which not possess a OMPT specialization (4.41 ± 2.96; *p* = 0.0003). However, more than half of the physiotherapists felt discouraged from using manual therapy to the neck region because of a perceived fear of causing adverse events (*n* = 267, 67.4%; 95%CI 62.8–72.0); that is, participants were highly concerned about vascular pathologies (mean = 7.7/10 points; SD = 2.2) and instability of the cranio-cervical junction (mean = 7.6/10 points; SD = 2.4). A significant difference was found in the perceived safety and working in a direct access setting for both vascular pathologies (7.97 ± 2.09; *p* = 0.004) and cervical instability (7.84 ± 2.33; *p* = 0.021) (Table 5). Although the majority of the respondents were aware of the lack of a causal link, they still had concerns mainly for spinal thrust manipulations (*n* = 270, 68.2%; 95%CI 63.6–72.8) or mobilizations (*n* = 110, 27.8%; 95%CI 23.4–32.2) compared to exercises (*n* = 21, 5.3%; 95%CI 3.1–7.5).

### 3.6. Training in Cranial Nerve Examination and Future Implications

Respondents considered having a specific training in CNE relevant (mean = 7.6/10 points; SD = 2.1); most of them considered that training in CNE should be provided during continuing professional development courses (*n* = 267, 67.4%; 95%CI 62.8–72.0) or within master’s programs (*n* = 166, 41.9%; 95%CI 37.1–46.8) with mixed theoretical and practical sessions (*n* = 363, 91.7%; 95%CI 88.9–94.4) for at least two days duration (*n* = 202, 51%; 95%CI 46.1–55.9).

## 4. Discussion

### 4.1. Key Findings

This is the first published study to investigate physiotherapists’ knowledge, understanding, and skills in the use of CNE, providing indications on future physiotherapy education, research, and practice. Our results highlight that CNE is considered relevant to be implemented in the screening of cervicocranial presentations and as part of the triage process. However, Italian physiotherapists reported not being sufficiently trained for an appropriate utilization in clinical practice. Interestingly, 57.8% of respondents stated that they had not received a specific training in CNE and linked this to omissions from the university’s core undergraduate curriculum programs (63.8%). A total of 31.0% of the not-trained did not consider CNE a relevant skill for their clinical practice. The most recent guidelines for the management of NAD recommend priority screening to rule out major pathologies mimicking musculoskeletal conditions [1,6,18,46]. Furthermore, it is the physiotherapists’ responsibility to screen if the patient’s presenting symptoms are within their scope of practice and appropriate for physiotherapy management. However, our findings show that although a large proportion of respondents worked in a direct (41.4%) access setting, most of them (64.6%) were not aware of guidelines when assessing patients with NAD with potential serious pathologies [1,5,21,47,48].

Clinicians should raise their index of suspicion of serious pathologies (i.e., congenital craniovertebral anomalies, cervical arterial pathologies, anatomical instabilities, autonomic disorders, etc.) during the subjective patient history taking. The identification of any red flags should be explored in detail with specific questioning as patients often did not think to mention them spontaneously [18]. It has been suggested that advanced clinical reasoning incorporating detailed knowledge of potential pathologies, combined with appropriate clinical testing (e.g., neurological signs or function examination), may be required to make the best informed judgement [23]. As clinicians cannot rely on valid and reliable screening tests that may help in identifying NAD IV patients [11], the neurological examination (i.e., cranial nerves, peripheral nerves, and upper motor neuron examination) is a key part of the triage process and may assist in evaluating the potential for serious conditions.

Although Italian physiotherapists occasionally encounter patients with potential concerning clinical presentations, such as headache, whiplash, neck or head trauma, dizziness, or visual disturbances, a relevant number (34.8%) of respondents did not routinely include CNE in their assessment, even when potentially required based on the patient’s history. Moreover, the majority suggested that they were not confident or secure (respectively, 38.4% and 37.1%) in conducting CN assessment, identifying pathognomonic signs and symptoms (respectively, 35.4% and 41.4%), or interpreting and managing the findings of the examination (respectively, 35.6% and 38.9%). Interestingly, those that had less clinical experience (<5 years) use the CNE significantly rarely and showed lesser confidence in interpreting the assessment finding. On the other hand, those possessing an OMPT specialization showed more confidence in conducting a CNE, interpreting the findings, and managing the examination results. This observation may be related to a significantly better understanding of the relevance of guidelines for the participants possessing an OMPT specialization. Although an increasing number of primary studies suggest the importance of CNE when examining the neck region to inform pattern recognition of sinister clinical conditions [13,47,49,50,51,52,53,54,55,56,57,58,59,60,61,62,63], our findings may reflect the lack of high-quality evidence for CN involvement in NAD IV. To the best of the authors’ knowledge, no specific data are available to support the diagnostic accuracy of a complete CNE. However, although peripheral neurological examination (e.g., sensory, motor, and reflex testing) has been shown to possess low sensitivity, moderate specificity, and limited diagnostic accuracy [49], the examination of isolated CN injury for focal impairment shows poor sensitivity (0.22) but high specificity (0.95) and is predictable for advanced diagnostic imaging [50], and raising the index of suspicion of serious pathologies. Therefore, it is suggested to contextualize the physical examination, including the neurological examination, with the clinical presentation and subjective patient history and to combine more tests in order to strengthen their clinical relevance [51].

Subtle transient neurological signs and symptoms, such as headache (81%), neck pain (57–80%), dizziness (32%), visual disturbance (34%), paresthesia (19–34%) [18], CN palsies [12,13,52,53,54,55,56,57,58], Horner’ syndrome, and tinnitus [54], are common predictors of potential serious pathologies (e.g., vascular pathologies) [18]. The cause of these conditions can be disabling or even lethal; therefore, understanding how to recognize, diagnose, and appropriately evaluate them is of great importance to all clinicians. CNE may assist in the identification of serious pathology when subtle transient neurological signs and symptoms are identified in neck pain patients [18].

Our study highlights the need for further education and research for an appropriate clinical utilization and diagnosis [51].

### 4.2. Recommendations for Clinical Practice

The results of our study show that Italian physiotherapists do not conduct an adequate screening for referral and systems review procedures. The lack of expertise and updated education in that field may explain the reported increased fear of manual therapy delivery to the neck because of perceived potential risk of adverse events (*n* = 267, 67.4%). That is, those without an OMPT specialization (*p* = 0.0003) and with more than 20 years of clinical experience (*p* = 0.001) showed a significant increase in belief that manual therapy to the neck can cause adverse events. It is important to note that the first IFOMPT Master’s program was started in 2004 (i.e., <20 years) [59], and physiotherapists that typically attend postgraduate programs are younger and have less experience than 20 years [60]. This observation may reflect the suspicion that many colleagues still based their clinical practice on continually propagated dogmatic knowledge instead of scientific clinical studies [61,62,63]. Therefore, we strongly encourage institutions and policymakers to use the findings of our study as a starting point to introduce appropriate screening for referral competencies into the Italian physiotherapy core curriculum and invite other research group to collaborate for further generalize our result in other countries.

Although there is no convincing evidence to support a causal link between spinal thrust manipulation and cervical artery dissection or anatomical instability [64], in addition to the notion that mobilization and manipulation have been shown to possess the same adverse events’ risk [65], the majority of Italian physiotherapists are more discouraged from using spinal thrust manipulation (68.2%) compared to mobilizations (27.8%) in patients with NAD. These anecdotal beliefs strongly influence physiotherapists’ clinical practice [65,66,67,68]. Therefore, we advise updating the knowledge of physiotherapists concerning adverse events. Furthermore, although cervical arterial dissection has been documented related to a wide variety exercises [18,69,70,71], physiotherapists are not accustomed to evaluating cardiovascular parameters [72,73,74], and only 5.3% of the respondents are concerned about the exercises’ related risk: considering that 62% of physiotherapists’ patients potentially have a history or suffer of cardiac disease, the risk of acute myocardial infarction during exercise is seven times higher than that of sudden cardiac death [75,76,77,78]. Therefore, we advise physiotherapists to consider risk factors and more specific cardiovascular parameters, in their clinical reasoning. To guide physiotherapists in their daily practice, we created an infographic decision tool for early identification of potential vascular/neurological pathologies of the neck for public use (Supplement 2). Moreover, an extensive description of the CNE is available elsewhere [79].

### 4.3. Strengths and Limitations

A key strength is the high response rate, which permitted a required sample size calculation, confirming the willingness of physiotherapists to participate in this study. Moreover, authors have adopted a previous local online survey to understand the opinion of the target population. The methodological choice was previously used in surveys representing a valid tool aimed to capture the perspective of a large sample of healthcare providers [80]. That is, although we do not send personal invitations, the publication of several reminders helped to recruit a number of Italian physiotherapists in line with previous surveys [44,45]. However, the number of responders could have been influenced by the detailed and specific questions that were employed. Additionally, there is also high potential for responder bias, as those with stronger positive or negative interest in the topic may be more likely to respond or to give more detail to the survey. The recruitment methodology may potentially lead to a selection bias.

## 5. Conclusions

Our study exposed a concerning number of Italian physiotherapists who work as first-line practitioners who had not been trained in the fundamentals of CNE. Many of those who had been trained reported a lack of knowledge or confidence about exactly when and how to implement CN screening. In addition, the physiotherapists surveyed in this study did not report confidence in identifying pathognomonic signs and symptoms of NAD IV, with a lack of clarity regarding exactly when to assess CNs. All the above may impact appropriate clinical reasoning and triage in such cases, having the potential to adversely impact on the patient and practitioner. We strongly encourage institutions and policymakers to use the findings of our study as a starting point to introduce appropriate screening for referral competencies into the Italian physiotherapy core curriculum.

### Highlights

- Triage of serious pathologies masquerading as neck pain before providing any evidence-based intervention is recommendation number one in clinical practice guidelines and a professional responsibility.
- Cranial nerve examination may potentially impact on appropriate clinical reasoning and the screening process for referral.
- It is concerning that a considerable number of Italian physical therapists who work as first line practitioners are not schooled in the fundamentals of cranial nerve examination.
- Improvement of the physiotherapy core curriculum concerning screening for referral competencies and cranial nerve examination is important.
- To guide physiotherapists in their daily practice, we created an infographic for public use.

## Figures and Tables

**Table 1:** Demographic and clinical characteristics of the sample.

Variables	N	%	95%CI
What is your highest earned degree?			
BSc	203	51.3	46.3–56.2
MSc	193	48.7	43.8–53.7
Did you earn an IFOMPT OMPT specialization?			
Yes	137	34.6	29.9–39.3
No	259	65.4	60.7–70.1
How many years have you been practicing as a licensed physical therapist?			
0–5	97	24.5	20.3–28.7
6–10	112	28.3	23.8–32.7
11–15	71	17.9	14.2–21.7
16–20	42	10.6	7.6–13.6
20+	74	18.7	14.8–22.5
What physical therapy setting(s) do you currently practice in? *			
Private practice (primary line care)	314	79.3	75.3–83.3
Hospital (secondary care line)	174	43.9	39.1–48.8
Education	34	8.6	5.8–11.3
Research	7	1.8	0.5–3.1
What main physical therapy access regimen do you practice in?			
Direct access	164	41.4	36.6–46.3
Secondary care referral pathway	232	58.6	53.7–63.4
How frequently do you assess patients with headache?			
Never	18	4.5	2.5–6.6
Rarely (1–5 patients yearly)	114	28.8	24.3–33.2
Occasionally (1–5 patients monthly)	175	44.2	39.3–49.1
Frequently (1–5 patients weekly)	79	19.9	16–23.9
Daily (>5 patients weekly)	10	2.5	1–4.1
How frequently do you assess patients with dizziness?			
Never	26	6.6	4.1–9
Rarely (1–5 patients yearly)	172	43.4	38.6–48.3
Occasionally (1–5 patients monthly)	152	38.4	33.6–43.2
Frequently (1–5 patients weekly)	41	10.4	7.4–13.4
Daily (>5 patients weekly)	5	1.3	0.2–2.4
How frequently do you assess patients with cervical/head trauma?			
Never	21	5.3	3.1–7.5
Rarely (1–5 patients yearly)	175	44.2	39.3–49.1
Occasionally (1–5 patients monthly)	143	36.1	31.4–40.8
Frequently (1–5 patients weekly)	46	11.6	8.5–14.8
Daily (>5 patients weekly)	11	2.8	1.2–4.4
How frequently do you assess patients with WAD?			
Never	21	5.3	3.1–7.5
Rarely (1–5 patients yearly)	171	43.2	38.3–48.1
Occasionally (1–5 patients monthly)	152	38.4	33.6–43.2
Frequently (1–5 patients weekly)	42	10.6	7.6–13.6
Daily (>5 patients weekly)	10	2.5	1–4.1

Abbreviations: %: percentage; CI: confidence interval; N: number; WAD: Whiplash and associated disorders. * Multiple choice close-ended questions.

**Table 2:** Response to each survey questions, summarized for IFOMPT OMPT specialization.

	Not Confident	Insecure	Quite Sure	Sure	*p*-Value *
Quantify your ability in conducting a cranial nerve examination					
IFOMPT OMPT specialization YES	35 (25.5%)	55 (40.1%)	44 (32.1%)	3 (2.2%)	<0.001
Adjusted residual	−3.8	0.9	3.0	1.2	
*p*-value **	0.0001	0.3648	0.0023	0.2294	
IFOMPT OMPT specialization NO	117 (45.2%)	92 (35.5%)	48 (18.5%)	2 (0.8%)	
Adjusted residual	3.8	−0.9	−3.0	−1.2	
*p*-value **	0.0001	0.3648	0.0023	0.2294	
Quantify your confidence in interpreting the findings within your cranial nerve examination					
IFOMPT OMPT specialization YES	32 (23.4%)	63 (46.0%)	40 (29.2%)	2 (1.5%)	<0.001
Adjusted residual	−3.6	1.3	2.2	1.9	
*p*-value **	0.0002	0.1792	0.0254	0.0512	
IFOMPT OMPT specialization NO	108 (41.7%)	101 (39.0%)	50 (19.3%)	0 (0.0%)	
Adjusted residual	3.6	−1.3	−2.2	−1.9	
*p*-value **	0.0002	0.1792	0.0254	0.0512	
Quantify your confidence in managing the findings within your cranial nerve examination					
IFOMPT OMPT specialization YES	32 (23.4%)	59 (43.1%)	43 (31.4%)	3 (2.2%)	<0.001
Adjusted residual	−3.7	1.2	2.2	2.4	
*p*-value **	0.0002	0.2149	0.0259	0.0168	
IFOMPT OMPT specialization NO	109 (42.1%)	95 (36.7%)	55 (21.2%)	0 (0.0%)	
Adjusted residual	3.7	−1.2	−2.2	−2.4	
*p*-value **	0.0002	0.2149	0.0259	0.0168	
	Rarely (1–5 Patients Yearly)	Occasionally (1–5 Patients Monthly)	Frequently (1–5 Patients Weekly)	Daily (>5 Patients Weekly)	*p*-Value *
If yes, how frequently do you use the cranial nerve examination?					
IFOMPT OMPT specialization YES	60 (58.8%)	29 (28.4%)	12 (11.8%)	1 (1.0%)	0.256 **
IFOMPT OMPT specialization NO	88 (56.4%)	57 (36.5%)	9 (5.8%)	2 (1.3%)	
			Yes	No	*p*-Value *
Do concerns about potential cervical adverse events discourage you from using manual therapy in the management of patients with cervical disorders?					
IFOMPT OMPT specialization YES			84 (61.3%)	53 (38.7%)	0.0591
IFOMPT OMPT specialization NO			183 (70.7%)	76 (29.3%)	
			Yes IFOMPT OMPT Specialization	No IFOMPT OMPT Specializationn	*p*-value ***
How valuable do you consider guidelines for the management of cervical disorders?			8.01 ± 1.91	7.34 ± 2.22	0.003
How relevant is cranial nerve examination to your practice?			7.69 ± 2.06	7.59 ± 2.00	0.655
To what extent do you consider cranial nerve examination relevant to cervical arterial pathologies?			7.71 ± 2.30	7.41 ± 2.23	0.205
To what extent are you concerned about cervical arterial pathologies when managing cervical disorders?			7.62 ± 2.16	7.75 ± 2.22	0.580
To what extent do you consider the cranial nerves examination relevant to pathologies of the cranio-cervical junction (e.g., ligament damage of the cranio-vertebral junction, cervical fracture, congenital anomalies, etc.)?			6.73 ± 2.64	6.67 ± 2.49	0.829
To what extent are you concerned about serious cervical pathologies (e.g., ligament damage of the cranio-vertebral junction, cervical fracture, congenital anomalies, etc.) when managing cervical disorders?			7.62 ± 2.48	7.60 ± 2.44	0.932
To what extent do you agree with the following sentence? “Manual therapy to the cervical spine can cause adverse events”			3.33 ± 2.69	4.41 ± 2.96	0.0003
How relevant do you consider training in cranial nerve examination?			7.69 ± 2.23	7.54 ± 2.14	0.495

Abbreviations: IFOMPT: International Federation of Orthopedic Manipulative Physical Therapists; OMPT: Orthopedic Manipulative Physical Therapist. Notes: Data were reported as frequency and percentage, or mean ± standard deviation. * referred to Chi-squared independent test. ** referred to adjusted residual (Bonferroni-corrected *p*-value = 0.00625). *** referred to independent *t*-test.

**Table 3:** Knowledge, education, and confidence with regard to cranial nerve examination.

Question	Mean (SD)	N (%)	95%CI
How valuable do you consider guidelines for the management of cervical disorders?			
Likert scale (0–10)	7.5 (2.1)		
Which international guidelines are you familiar with *			
IFOMPT cervical arterial dysfunction framework		88 (22.2)	18.1–26.3
NICE headache assessment clinical knowledge summary		62 (15.7)	12.1–19.2
Nottingham cervical arterial dysfunction classification model		27 (6.8)	4.3–9.3
None		256 (64.6)	59.9–69.4
Have you received training in cranial nerve examination?			
Yes		167 (42.2)	37.3–47.0
No		229 (57.8)	53.0–62.7
If yes, where did you learn cranial nerve examination? *			
Workplace		22 (13.2)	8.0–18.3
Continuing Professional Development courses		77 (46.1)	38.5–53.7
During the Bachelor		50 (29.9)	23.0–36.9
During the Master		73 (43.7)	36.2–51.2
Interaction with other healthcare professionals		44 (26.3)	19.7–33.0
Personal readings (scientific books or literature)		83 (49.7)	44.8–54.6
Social media and podcast		15 (9.0)	4.6–13.3
If no, why are you not interested in it?			
Not relevant for my practice		71 (31.0)	25.0–37.0
Outside the physical therapy’s scope		6 (2.6)	0.6–4.7
Working in secondary care referral pathway		6 (2.6)	0.6–4.7
Lack of education		146 (63.8)	57.5–70.0
How relevant is cranial nerve examination to your practice?			
Likert scale (0–10)	7.6 (2.0)		
Do you use the cranial nerve examination in your practice?			
No		138 (34.8)	30.2–39.5
Yes		258 (65.2)	60.5–69.8
If no, why?			
Outside the physical therapy scope of practice		34 (24.6)	17.4–31.8
Working in a secondary care referral pathway (patients previously evaluated by a physician)		9 (6.5)	2.4–10.6
Not trained adequately		94 (68.1)	60.3–75.9
Requires too much time		1 (0.7)	0.7–2.1
If yes, how frequently do you use the cranial nerve examination?			
Rarely (1–5 patients yearly)		148 (57.4)	51.3–63.4
Occasionally (1–5 patients monthly)		86 (33.3)	27.6–39.1
Frequently (1–5 patients weekly)		21 (8.1)	4.8–11.5
Daily (>5 patients weekly)		3 (1.2)	0.0–2.5
What anamnestic items would prompt you to use the cranial nerve examination? **			
Neck/head trauma		77 (29.8)	24.2–35.4
Dizziness		87 (33.7)	27.9–39.4
Headache		78 (30.2)	24.6–35.8
Drop attack		7 (2.7)	0.7–4.6
Visual disturbances		101 (39.1)	33.1–45.1
Nausea		40 (15.5)	11.0–19.9
Cardiovascular symptoms		23 (8.9)	5.4–12.3
Nystagmus		56 (21.7)	16.6–26.7
5D & 3N		10 (3.9)	1.5–6.2
Tinnitus		41 (15.9)	11.4–20.3
Pain		101 (39.1)	33.1–45.1
Dysphagia		33 (12.8)	8.7–16.8
Dysarthria		13 (5.0)	2.3–7.7
Diplopia		70 (27.1)	21.7–32.5
Paresthesia		82 (31.8)	26.1–37.4
Sensitivity deficit		88 (34.1)	28.3–39.8
Balance deficit		40 (15.5)	11.0–19.9
Movement deficit		61 (23.6)	18.4–28.8
Cognitive alterations		9 (3.5)	1.2–5.7
Quantify your ability in conducting a cranial nerve examination			
Not confident		152 (38.4)	33.6–43.2
Insecure		147 (37.1)	32.4–41.9
Quite sure		92 (23.2)	19.1–27.4
Sure		5 (1.3)	0.2–2.4
Quantify your confidence in interpreting the findings within your cranial nerve examination			
Not confident		140 (35.4)	30.6–40.1
Insecure		164 (41.4)	36.6–46.3
Quite sure		90 (22.7)	18.6–26.9
Sure		2 (0.5)	0.0–1.2
Quantify your confidence in managing the findings within your cranial nerve examination			
Not confident		141 (35.6)	30.9–40.3
Insecure		154 (38.9)	34.1–43.7
Quite sure		98 (24.7)	20.5–29.0
Sure		3 (0.8)	0.0–1.6
How do you manage abnormal findings during the cranial nerve examination? *			
Monitoring patient’s symptoms		128 (32.3)	27.7–36.9
Refer to general practitioner		184 (46.5)	41.6–51.4
Referral to the Emergency Department		66 (16.7)	13.0–20.3
Referral to a specialist		268 (67.7)	63.1–72.3
Request further examination		53 (13.4)	10.0–16.7
To what extent do you consider cranial nerve examination relevant to cervical arterial pathologies?			
Likert scale (0–10)	7.5 (2.2)		
To what extent are you concerned about cervical arterial pathologies when managing cervical disorders?			
Likert scale (0–10)	7.7 (2.2)		
To what extent do you consider the cranial nerve examination relevant to pathologies of the cranio-cervical junction (e.g., ligament damage of the cranio-vertebral junction, cervical fracture, congenital anomalies, etc.)?			
Likert scale (0–10)	6.7 (2.5)		
To what extent are you concerned about serious cervical pathologies (e.g., ligament damage of the cranio-vertebral junction, cervical fracture, congenital anomalies, etc.) when managing cervical disorders?			
Likert scale (0–10)	7.6 (2.4)		
Do concerns about potential cervical adverse events discourage you from using manual therapy in the management of patients with cervical disorders?			
Yes		267 (67.4)	62.8–72.0
No		129 (32.6)	28.0–37.2
To what extent do you agree with the following sentence? “Manual therapy to the cervical spine can cause adverse events”			
Likert scale (0–10)	4.0 (2.9)		
What therapeutic interventions do you consider dangerous and capable of worsening or causing adverse events (e.g., cervical arterial dissection)? *			
HVLA thrust manipulation		270 (68.2)	63.6–72.8
Mobilization		110 (27.8)	23.4–32.2
Soft tissue techniques (e.g., massage)		36 (9.1)	6.3–11.9
Exercises		21 (5.3)	3.1–7.5
Modalities		62 (15.7)	12.1–19.2
None		66 (16.7)	13.0–20.3
Do you use other screening procedures to screen (triage) serious cervical pathologies? *			
Canadian Cervical Spine Rules		32 (8.1)	5.4–10.8
Pre-Manipulative testing (e.g., Sharp Purser test, Alar lig. test, Anterior drawer test, etc.)		176 (44.4)	39.6–49.3
History items (e.g., 5D & 3Ns)		46 (11.6)	8.5–14.8
Imaging (Xray, CT scan, MRI)		51 (12.9)	9.6–16.2
None		91 (23.0)	18.8–27.1
How relevant do you consider training in cranial nerve examination?			
Likert scale (0–10)	7.6 (2.1)		
How should training in conducting cranial nerve examination be provided? *			
Within postgraduate programs (Masters)		166 (41.9)	37.1–46.8
Within the undergraduate programs (Bachelor)		281 (71.0)	66.5–75.4
Within Continuing Professional Development courses		267 (67.4)	62.8–72.0
In the work place		87 (22.0)	17.9–26.0
What should training of cranial nerve examination consist of?			
Practical		26 (6.6)	4.1–9–0
Theoretical		7 (1.8)	0.5–3.1
Mixed		363 (91.7)	88.9–94.4
What duration should training of cranial nerve examination have?			
Half day		23 (5.8)	3.5–8.1
1 day		81 (20.5)	16.5–24.4
2 days		202 (51.0)	46.1–55.9
1 week		69 (17.4)	13.7–21.2
>1 week		21 (5.3)	3.1–7.5

Abbreviations: %: percentage; CI: confidence interval; N: number; SD: Standard Deviation; IFOMPT: International Federation of Orthopedic Manipulative Physical Therapists; NICE, National Institute for Health and Care Excellence; 5D & 3N: dysphagia, diplopia, drop attack, dizziness and dysarthria & numbness, nystagmus and nausea; CT: computed tomography; MRI: Magnetic Resonance Imaging. * Multiple choice close-ended questions; N: number. ** Open-ended question.

**Table 4:** Response to each survey questions, summarized for years’ experience.

		Not Confident	Insecure	Quite Sure	Sure	*p*-Value *
Quantify your ability in conducting a cranial nerve examination						
0–5 years of practice as a licensed PT		45 (46.4%)	41 (42.3%)	11 (11.3%)	0 (0.0%)	0.1237
6–10 years of practice as a licensed PT		38 (33.9%)	45 (40.2%)	27 (24.1%)	2 (1.8%)	
11–15 years of practice as a licensed PT		24 (33.8%)	25 (35.2%)	20 (28.2%)	2 (2.8%)	
16–20 years of practice as a licensed PT		15 (35.7%)	13 (31.0%)	13 (31.0%)	1 (2.4%)	
20+ years of practice as a licensed PT		30 (40.5%)	23 (31.1%)	21 (28.4%)	0 (0.0%)	
Quantify your confidence in interpreting the findings within your cranial nerve examination						
0–5 years of practice as a licensed PT		37 (38.1%)	49 (50.5%)	11 (11.3%)	0 (0.0%)	0.0128
Adjusted residual		0.7	2.1	−3.1	−0.8	
*p*-value **		0.5081	0.0362	0.0020	0.4193	
6–10 years of practice as a licensed PT		40 (35.7%)	48 (42.9%)	24 (21.4%)	0 (0.0%)	
Adjusted residual		0.1	0.4	−0.4	−0.9	
*p*-value **		0.9248	0.7142	0.6985	0.3732	
11–15 years of practice as a licensed PT		17 (23.9%)	33 (46.5%)	20 (28.2%)	1 (1.4%)	
Adjusted residual		−2.2	1.0	1.2	1.2	
*p*-value **		0.0264	0.3388	0.2271	0.2358	
16–20 years of practice as a licensed PT		15 (35.7%)	12 (28.6%)	14 (33.3%)	1 (2.4%)	
Adjusted residual		0.1	−1.8	1.7	1.8	
*p*-value **		0.9587	0.0739	0.0827	0.0696	
20+ years of practice as a licensed PT		31 (41.9%)	22 (29.7%)	21 (28.4%)	0 (0.0%)	
Adjusted residual		1.3	−2.3	1.3	−0.7	
*p*-value **		0.1919	0.0236	0.1982	0.4967	
Quantify your confidence in managing the findings within your cranial nerve examination						
0–5 years of practice as a licensed PT		39 (40.2%)	43 (44.3%)	15 (15.5%)	0 (0.0%)	0.1674
6–10 years of practice as a licensed PT		39 (34.8%)	44 (39.3%)	29 (25.9%)	0 (0.0%)	
11–15 years of practice as a licensed PT		17 (23.9%)	32 (45.1%)	21 (29.6%)	1 (1.4%)	
16–20 years of practice as a licensed PT		15 (35.7%)	13 (31.0%)	13 (31.0%)	1 (2.4%)	
20+ years of practice as a licensed PT		31 (41.9%)	22 (29.7%)	20 (27.0%)	1 (1.4%)	
If yes, how frequently do you use the cranial nerve examination?						
0–5 years of practice as a licensed PT		44 (80.0%)	11 (20.0%)	0 (0.0%)	0 (0.0%)	0.0453
Adjusted residual		3.8	−2.4	−2.5	−0.9	
*p*-value **		0.0001	0.0180	0.0128	0.3644	
6–10 years of practice as a licensed PT		41 (56.2%)	25 (34.2%)	6 (8.2%)	1 (1.4%)	
Adjusted residual		−0.2	0.2	0.02	0.2	
*p*-value **		0.8065	0.8450	0.9765	0.8454	
11–15 years of practice as a licensed PT		26 (52.0%)	19 (38.0%)	4 (8.0%)	1 (2.0%)	
Adjusted residual		−0.9	0.8	0.04	0.6	
*p*-value **		0.3929	0.4356	0.9679	0.5385	
16–20 years of practice as a licensed PT		16 (51.6%)	10 (32.3%)	5 (16.1%)	0 (0.0%)	
Adjusted residual		−0.7	−0.1	1.7	−0.6	
*p*-value **		0.4899	0.8922	0.0828	0.5196	
20+ years of practice as a licensed PT		21 (42.9%)	21 (42.9%)	6 (12.2%)	1 (2.0%)	
Adjusted residual		−2.3	1.6	1.2	0.6	
*p*-value **		0.0225	0.1161	0.2429	0.5241	
				Yes	No	*p*-Value ***
Do concerns about potential cervical adverse events discourage you from using manual therapy in the management of patients with cervical disorders?						
0–5 years of practice as a licensed PT				68 (70.1%)	29 (29.9%)	0.0652
6–10 years of practice as a licensed PT				65 (58.0%)	47 (42.0%)	
11–15 years of practice as a licensed PT				54 (76.1%)	17 (23.9%)	
16–20 years of practice as a licensed PT				26 (61.9%)	16 (38.1%)	
20+ years of practice as a licensed PT				54 (73.0%)	20 (27.0%)	
	0–5 Years of Practice as a Licensed PT	6–10 Years of Practice as a Licensed PT	11–15 Years of Practice as a Licensed PT	16–20 Years of Practice as a Licensed PT	20+ Years of Practice as a Licensed PT	*p*-Value ***
How valuable do you consider guidelines for the management of cervical disorders?	7.85 ± 1.86	7.66 ± 2.04	7.48 ± 2.09	7.57 ± 2.17	7.16 ± 2.63	0.332
How relevant is cranial nerve examination to your practice?	7.46 ± 2.09	7.89 ± 1.79	7.42 ± 2.16	7.48 ± 2.13	7.70 ± 2.08	0.461
To what extent do you consider cranial nerves examination relevant to cervical arterial pathologies?	7.42 ± 2.22	7.59 ± 2.25	7.34 ± 2.25	7.36 ± 2.36	7.76 ± 2.30	0.780
To what extent are you concerned about cervical arterial pathologies when managing cervical disorders?	7.64 ± 1.96	7.65 ± 2.34	7.73 ± 2.22	7.24 ± 2.46	8.11 ± 2.06	0.337
To what extent do you consider the cranial nerves examination relevant to pathologies of the cranio-cervical junction (e.g., ligament damage of the cranio-vertebral junction, cervical fracture, congenital anomalies, etc.)?	6.45 ± 2.28	6.64 ± 2.55	6.87 ± 2.60	6.31 ± 2.82	7.12 ± 2.62	0.365
To what extent are you concerned about serious cervical pathologies (e.g., ligament damage of the cranio-vertebral junction, cervical fracture, congenital anomalies, etc.) when managing cervical disorders?	7.36 ± 2.53	7.63 ± 2.16	7.83 ± 2.41	7.02 ± 2.84	8.01 ± 2.51	0.199
To what extent do you agree with the following sentence? “Manual therapy to the cervical spine can cause adverse events”	3.64 ± 2.54	3.66 ± 2.97	4.17 ± 2.91	3.50 ± 2.58	5.31 ± 3.15	**0.001**

Abbreviations: PT: physiotherapist. Notes: significant *p*-values are reported in bold. * referred to Chi-squared independent test. ** referred to adjusted residual (Bonferroni-corrected *p*-value = 0.00625). *** referred to univariate ANOVA.

**Table 5:** Response to each survey questions, summarized for physiotherapy access regimen.

	Not Confident	Insecure	Quite Sure	Sure	*p*-Value *
Quantify your ability in conducting a cranial nerve examination					
Direct access	90 (38.8%)	81 (34.9%)	56 (24.1%)	5 (2.2%)	0.2130
Secondary care referral pathway	62 (37.8%)	66 (40.2%)	36 (22.0%)	0 (0.0%)	
Quantify your confidence in interpreting the findings within your cranial nerve examination					
Direct access	85 (36.6%)	87 (37.5%)	58 (25.0%)	2 (0.9%)	0.1702
Secondary care referral pathway	55 (33.5%)	77 (47.0%)	32 (19.5%)	0 (0.0%)	
Quantify your confidence in managing the findings within your cranial nerve examination					
Direct access	82 (35.3%)	81 (34.9%)	67 (28.9%)	2 (0.9%)	0.1008
Secondary care referral pathway	59 (36.0%)	73 (44.5%)	31 (18.9%)	1 (0.6%)	
	Rarely (1–5 Patients Yearly)	Occasionally (1–5 Patients Monthly)	Frequently (1–5 Patients Weekly)	Daily (>5 Patients Weekly)	*p*-Value *
If yes, how frequently do you use the cranial nerve examination?					
Direct access	82 (55.4%)	50 (33.8%)	15 (10.1%)	1 (0.7%)	0.4470
Secondary care referral pathway	66 (60.0%)	36 (32.7%)	6 (5.5%)	2 (1.8%)	
			Yes	No	*p*-Value *
Do concerns about potential cervical adverse events discourage you from using manual therapy in the management of patients with cervical disorders?					
Direct access			158 (68.1%)	74 (31.9%)	0.7315
Secondary care referral pathway	109 (66.5%)			55 (33.5%)	
			Direct Access	Secondary Care referral Pathway	*p*-Value **
How valuable do you consider guidelines for the management of cervical disorders?			7.77 ± 2.09	7.29 ± 2.19	0.030
How relevant is cranial nerve examination to your practice?			7.75 ± 2.05	7.45 ± 1.97	0.154
To what extent do you consider cranial nerves examination relevant to cervical arterial pathologies?			7.63 ± 2.33	7.34 ± 2.15	0.212
To what extent are you concerned about cervical arterial pathologies when managing cervical disorders?			7.97 ± 2.09	7.33 ± 2.29	0.004
To what extent do you consider the cranial nerves examination relevant in pathologies of the cranio-cervical junction (e.g., ligament damage of the cranio-vertebral junction, cervical fracture, congenital anomalies, etc.)?			6.63 ± 2.64	6.78 ± 2.40	0.560
To what extent are you concerned about serious cervical pathologies (e.g., ligament damage of the cranio-vertebral junction, cervical fracture, congenital anomalies, etc.) when managing cervical disorders?			7.84 ± 2.33	7.27 ± 2.58	0.021
To what extent do you agree with the following sentence? “Manual therapy to the cervical spine can cause adverse events”			4.00 ± 2.91	4.09 ± 2.92	0.785
How relevant do you consider training in cranial nerve examination?			7.72 ± 2.09	7.40 ± 2.27	0.147

Notes: * referred to Chi-squared independent test. ** referred to univariate ANOVA.

## Data Availability

Raw data are available upon request.

## Supplementary Materials

The following are available online at https://www.mdpi.com/article/10.3390/healthcare9101262/s1, Supplement 1: Survey Questions, Supplement 2: Infographic Decision Tool for early identification of potential vascular/neurological pathologies of the neck.
