# Supplementary material for: Assessing Cranial Nerves in Physical Therapy Practice: Findings from a Cross-Sectional Survey and Implication for Clinical Practice
Source: Healthcare (Basel). 2021 Sep 24;9(10):1262. doi: 10.3390/healthcare9101262 (PMC8535196; doi:10.3390/healthcare9101262)
Supplement: Supplementary file 1 [file healthcare-09-01262-s001.zip › Supplement 1 CNE.pdf]

Supplement 1. Survey Questions: *Multiple choice close-ended questions; **Open ended question.

About you

Are you a physiotherapist practicing in Italy? If you are not practicing in Italy please select NO, to exit the questionnaire
Yes
No

This information will be used to compare different groups of physiotherapists
What is your highest earned degree?
BSc
MSc
PhD
Did you earn an IFOMPT OMPT specialization?
Yes
No
How many years have you been practicing as a licensed physical therapist?
0-5
6-10
11-15
16-20
20+
What physical therapy setting(s) do you currently practice in? *
Private practice (primary line care)
Hospital (secondary care line)
Education
Research
What main physical therapy access regimen do you practice in?
Direct access
Secondary care referral pathway
How frequently do you assess patients with headache?
Never
Rarely (1-5 patients yearly)
Occasionally (1-5 patients monthly)
Frequently (1-5 patients weekly)
Daily (>5 patients weekly)
How frequently do you assess patients with dizziness?
Rarely (1-5 patients yearly)
Occasionally (1-5 patients monthly)
Frequently (1-5 patients weekly)
Daily (>5 patients weekly)
How frequently do you assess patients with cervical/head trauma?
Rarely (1-5 patients yearly)
Occasionally (1-5 patients monthly)
Frequently (1-5 patients weekly)
Daily (>5 patients weekly)
How frequently do you assess patients with WAD?
Rarely (1-5 patients yearly)
Occasionally (1-5 patients monthly)
Frequently (1-5 patients weekly)
Daily (>5 patients weekly)

KNOWLEDGE OF GUIDELINES RELEVANT TO CRANIAL NERVE EXAMINATION

How valuable do you consider guidelines for the management of cervical disorders?
Likert scale (0-10)

Which international guidelines are you familiar with*
IFOMPT cervical arterial dysfunction framework
NICE headache assessment clinical knowledge summary
Notthingham cervical arterial dysfunction classification model
None

EDUCATION IN CRANIAL NERVE EXAMINATION
Have you received training in cranial nerve examination?
Yes
No
If yes, where did you learn cranial nerve examination? *
Workplace
Continuing Professional Development courses
During the Bachelor
During the Master
Interaction with other healthcare professionals
Personal readings (scientific books or literature)
Social media and podcast
If no, why are you not interested in?
Not relevant for my practice
Outside the physical therapy's scope
Working in secondary care referral pathway
Lack of education

ABOUT THE USE OF THE CRANIAL NERVE EXAMINATION
How relevant is cranial nerve examination in your practice?
Likert scale (0-10)
Do you use the cranial nerves examination in your practice?
Yes
No
If no, why?
Outside the physical therapy scope of practice
Working in a secondary care referral pathway (patients previously evaluated by a physician)
Not trained adequately
Requires too much time
If yes, how much frequently do you use the cranial nerve examination?
Rarely (1-5 patients yearly)
Occasionally (1-5 patients monthly)
Frequently (1-5 patients weekly)
Daily (>5 patients weekly)
What anamnestic items would prompt you to use the cranial nerve examination? **
Neck/Head trauma
Dizziness
Headache
Drop attack
Visual disturbances
Nausea
Cardiovascular symptoms
Nystagmus
5D & 3N
Tinnitus
Pain
Dysphagia
Dysarthria
Diplopia

Paresthesia
Sensitivity deficit
Balance deficit
Movement deficit
Cognitive alterations

CONFIDENCE USING CRANIAL NERVE EXAMINATION
Quantify your ability in conducting a cranial nerve examination
Not confident
Insecure
Quite sure
Sure
Quantify your confidence in interpreting the findings within your cranial nerve examination
Not confident
Insecure
Quite sure
Sure
Quantify your confidence in managing the findings within your cranial nerve examination
Not confident
Insecure
Quite sure
Sure
How do you manage abnormal findings during the cranial nerve examination? *
Monitoring patient's symptoms
Refer to general practitioner
Referral to the Emergency Department
Referral to a Specialist
Request further examination

ATTITUDES TOWARDS CERVICAL SERIOUS CONDITIONS
To what extent do you consider cranial nerves examination relevant in cervical arterial pathologies?
Likert scale (0-10)
To what extent are you concerned about cervical arterial pathologies when managing cervical disorders?
Likert scale (0-10)
To what extent do you consider the cranial nerves examination relevant in pathologies of the cranio-cervical junction (e.g. ligament damage of the cranio-vertebral junction, cervical fracture, congenital anomalies, etc.)?
Likert scale (0-10)
To what extent are you concerned about serious cervical pathologies (e.g. ligament damage of the cranio-vertebral junction, cervical fracture, congenital anomalies, etc.) when managing cervical disorders?
Likert scale (0-10)
Does concerns about potential cervical adverse events discourage you from using manual therapy in the management of patients with cervical disorders?
Yes
No
To what extent do you agree with the following sentence? "Manual therapy to the cervical spine can cause adverse events"
Likert scale (0-10)
What therapeutic interventions do you consider dangerous and capable of worsening or causing adverse events (e.g. cervical arterial dissection)? *
HVLA thrust manipulation
Mobilization
Soft tissue techniques (e.g. massage)
Exercises
Modalities

None

<u>Do you use other screening procedures to screen (triage) serious cervical pathologies? *</u>

Canadian Cervical Spine Rules

Pre-Manipulative testing (e.g. vertebrobasilar insufficiency, ligamentous instability, etc)

History items (e.g. 5D & 3Ns)

Imaging (Xray, CT scan, MRI)

None

TRAINING IN CRANIAL NERVE ASSESSMENT

<u>How much relevant do you consider training in cranial nerve examination?</u>

Likert scale (0-10)

<u>How training in conducting cranial nerve examination should be provided? *</u>

Within the under-graduate programs (Bachelor)

Within post-graduate programs (Masters)

Within Continuing Professional Development courses

In the workplace

<u>What should training of cranial nerve examination consist of?</u>

Practical

Theoretical

Mixed

<u>What duration should training of cranial nerve examination have?</u>

half day

1 day

2 days

1 week

> 1 week
