# Supplementary material for: Assessing Cranial Nerves in Physical Therapy Practice: Findings from a Cross-Sectional Survey and Implication for Clinical Practice
Source: Healthcare (Basel). 2021 Sep 24;9(10):1262. doi: 10.3390/healthcare9101262 (PMC8535196; doi:10.3390/healthcare9101262)
Supplement: Supplementary file 1 [file healthcare-09-01262-s001.zip › Supplement 2.pdf]

DECISION TOOL FOR EARLY IDENTIFICATION OF POTENTIAL VASCULAR/NEUROLOGICAL PATHOLOGIES OF THE NECK

Decide your clinical action based on the level of concern

LEVEL OF CONCERN

LOW → HIGH

NO CONCERNING FEATURES

Revise management if clinical features change unexpectedly. Also, attention must be paid to specific questioning for and recognise transient antecedent neurological subtle signs and symptoms. Acute onset of pain described "unlike any other" must rise the clinician suspicion.
Notably, early presentation clinically present with non-ischemic symptoms (mimicking musculoskeletal symptoms) that can precede ischemic events by few days to several weeks.

LOW RISK PROFILE FEW CONCERNING FEATURES

Treat & monitor progress closely (vigilance).
However, a further extrinsic or environmental risk factors evaluation is advised especially in younger patients (< 45 years) for dissection vascular events. Also related, cardiovascular risk factor (especially for atherosclerosis) assessment is advised in older patients (> 45 years) for non-dissection vascular events (e.g. trombotic stroke).

MODERATE RISK PROFILE SOME CONCERNING FEATURES

DO NOT treat.
URGENT referral in warranted.
Probable pre-ischemia features:

- Transient antecedent neurological signs and symptoms (most common are visual disturbance, dizziness and imbalance).
- Vascular somatic symptoms (pain localization).
- Autonomic signs and symptoms (Horner's syndrome, especially ptosis)
- Cranial nerve palsy.

HIGH RISK PROFILE CONCERNING FEATURES

DO NOT treat EMERGENCY referral is warranted.
Transient brain ischemia features:

- For dissection on the posterior (vertebrobasilar) system (from most to least common) Unsteadiness/ataxia, Dysphasia/dysarthria/aphasia, Weakness of the Lower Limb.
- For dissection on the anterior (internal carotid artery) system (from most to least common) Weakness of the Upper Limb, Facial palsy, Dysphasia/dysarthria/aphasia.
- For non-dissection vascular events (from most to least common) hemiparesis, speech disturbance, balance disturbance.

EMERGENCY PROFILE

Hospitalization of the patient. Having this kind of patient in a physiotherapy setting is an exception.

Late-ischemia:

- For non-dissection stroke neurological features will depend on the area of the brain supplied by the dissected artery.
- Hind brain, (brainstem, cerebellum and posterior cortex) stroke for dissection on the posterior (vertebrobasilar) system.
- Cerebral and retinal stroke for dissection on the anterior (internal carotid artery) system.

No concerning features	Low risk profile	Moderate risk profile	High risk profile
Note that neck pain or headache + acute onset described as "never complained before/unusual" + transient preceding neurological signs are highly predictive for dissection vascular event.	Dissection related risk for Younger patients (< 45 years): recent trauma, vascular anomaly, recent infection (especially respiratory), and viral illness which imply valsalva activities.	Cranial Nerve Examination:<br>- Cranial nerve palsy is most commonly involved following dissection of the posterior cervical artery system.<br>- Lower Cranial nerves (V, VI, VIII, IX, X, XII) are most commonly involved.	In older patients, measuring blood pressure may be considered, since hypertension is a recognised risk factor for both non-dissection stroke and cardiovascular disease.
	Non-dissection related risk for older patiens (> 45 years): current or past smoker, hypertension, high colesterol and atherosclerosis.	Transient antecedent neurological signs & symptoms are often subtle and transient and may not be recognised as significant by either patient or clinician and may be present for days or weeks prior to the dissection.	There is a positive correlation between increased systolic and diastolic pressure and risk of stroke, which is the higher the pressure, the greater the risk.
		Autonomic signs & symptoms such as Horner's syndrome (especially ptosis) strongly indicate a peripheral compression due to the increased caliber of the artery mainly because an aneurism. Palpation of the artery may be helpfull.	Upper motor neuron examination may be helpfull.

LEVEL OF BLOOD PRESSURE	SYSTOLIC BLOOD PRESSURE (mmHg)		DIASTOLIC BLOOD PRESSURE (mmHg)
NORMAL	<130	and	<85
NORMAL HIGH BLOOD PRESSURE	130-139	and/or	85-89
GRADE 1 HYPERTENSION	140-159	and/or	90-99
GRADE 2 HYPERTENSION	>160	and/or	>100

A

REFERENCES

1. Rushton, A., Carlesso, L.C., Flynn, T., Hing, W.A., Kerry, R., Rubinstein, S.M., Vogel, S., 2020. International Framework for Examination of the Cervical Region for Potential of Vascular Pathologies of the Neck Prior to Orthopaedic Manual Therapy (OMT) Intervention: International IFOMPT Cervical Framework.
2. Unger, T., et al., 2020 International Society of Hypertension Global Hypertension Practice Guidelines. Hypertension, 2020. 75.
3. Matsumoto, H., Hanayama, H., Sakurai, Y., Minami, H., Masuda, A., Tominaga, S., Miyaji, K., Yamaura, I., Yoshida, Y., Hirata, Y., 2019. Investigation of the characteristics of headache due to unruptured intracranial vertebral artery dissection. Cephalalgia 39, 504–514.
4. Hutting, N., Kerry, R., Kranenburg, H., Mourad, F., Taylor, A., 2021. Assessing Vascular Function in Patients with Neck Pain, Headache and/or Orofacial Pain: part of the Job Description of all Physical Therapists. J Orthop Sports Phys Ther. In press.
5. Kerry, R., Taylor, A.J., 2009. Cervical arterial dysfunction: knowledge and reasoning for manual physical therapists. J. Orthop. Sports Phys. Ther. 39, 378–387.
6. Taylor, A., Mourad, F., Kerry, R., Hutting, N., 2021. A guide to cranial nerve testing for musculoskeletal clinicians. Journal of Manual & Manipulative Therapy. Submitted for publication
7. Sizer Jr PS, Brismée J-M, Cook C. Medical screening for red flags in the diagnosis and management of musculoskeletal spine pain. Pain Pract. 2007;7(1):53–71.
8. Hutting, N., Kranenburg, H.A., Rik, ", Kerry, R., 2020. Yes, we should abandon pretreatment positional testing of the cervical spine. Musculoskelet. Sci. Pract. 49, 102181.
